# Transcriptome Analysis of *Syringa oblata* Lindl. Inflorescence Identifies Genes Associated with Pigment Biosynthesis and Scent Metabolism

**DOI:** 10.1371/journal.pone.0142542

**Published:** 2015-11-20

**Authors:** Jian Zheng, Zenghui Hu, Xuelian Guan, Dequan Dou, Guo Bai, Yu Wang, Yingtian Guo, Wei Li, Pingsheng Leng

**Affiliations:** 1 College of Landscape Architecture, Beijing University of Agriculture, Beijing, 102206, China; 2 Beijing Engineering Research Center of rural landscape planning and design, Beijing, 102206, China; 3 College of Landscape Architecture and Forestry, Qingdao Agricultural University, Qingdao, 266100, China; Huazhong University of Science and Technology, CHINA

## Abstract

*Syringa oblata* Lindl. is a woody ornamental plant with high economic value and characteristics that include early flowering, multiple flower colors, and strong fragrance. Despite a long history of cultivation, the genetics and molecular biology of *S*. *oblata* are poorly understood. Transcriptome and expression profiling data are needed to identify genes and to better understand the biological mechanisms of floral pigments and scents in this species. Nine cDNA libraries were obtained from three replicates of three developmental stages: inflorescence with enlarged flower buds not protruded, inflorescence with corolla lobes not displayed, and inflorescence with flowers fully opened and emitting strong fragrance. Using the Illumina RNA-Seq technique, 319,425,972 clean reads were obtained and were assembled into 104,691 final unigenes (average length of 853 bp), 41.75% of which were annotated in the NCBI non-redundant protein database. Among the annotated unigenes, 36,967 were assigned to gene ontology categories and 19,956 were assigned to eukaryoticorthologous groups. Using the Kyoto Encyclopedia of Genes and Genomes pathway database, 12,388 unigenes were sorted into 286 pathways. Based on these transcriptomic data, we obtained a large number of candidate genes that were differentially expressed at different flower stages and that were related to floral pigment biosynthesis and fragrance metabolism. This comprehensive transcriptomic analysis provides fundamental information on the genes and pathways involved in flower secondary metabolism and development in *S*. *oblata*, providing a useful database for further research on *S*. *oblata* and other plants of genus *Syringa*.

## Introduction

Color and scent are important properties of flowers and play an important role in the ecophysiology, aesthetic properties, and economic value of flowering plants. Each plant possesses a unique and distinct floral color and scent. Exploring the generation mechanism for floral color and scent is necessary to reveal their roles in plants, and to breed new varieties through regulation of color and scent.

The diversity of floral color results from the difference in flower pigments. Flower pigments vary among plant species according to the characteristics of their low-molecular-mass secondary metabolites, such as flavonoids, carotenoids, and alkaloids, of which flavonoids are the dominant compounds[1]. The flavonoid biosynthetic pathway has been thoroughly studied in model plants such as *Arabidopsis thaliana*, *petunia spp*., *Antirrhinum majus*, *Vitis vinifera*, and *Zea mays*[2–4]. Anthocyanidins (including pelargonidin, cyanidin, delphinidin, peonidin, petunidin, and malvidin) in floral organs are the main flavonoids that determine flower color. The regulatory mechanisms of anthocyanin metabolism have been resolved in some ornamental plants, such as *Gerbera hybrida*, *Gentian scabra*, *Lilium* spp., and *P*. *hybrida*[5–9]. Anthocyanidins and many of the related biosynthetic enzymes [e.g., Chalcone synthase (CHS), chalcone-flavanone isomerase (CHI), flavanone 3-hydroxylase (F3H), flavonoid-3′-hydroxylase (F3′H), flavonoid 3′5′-hydroxylase (F3′5′H), dihydroflavonol 4-reductase (DFR), anthocyanidin synthase (ANS), glycosyltransferase (GT), acyltransferase (AT), and methyltransferase (MT)] have been identified and functionally characterized[3]. The different biosynthesis and regulatory mechanisms of anthocyanidins found in different plants should be explored.

Floral scents are composed of volatile compounds, of which terpenoids, phenylpropanoids/benzenoids, and fatty-acid derivatives are the main components. The floral scents of ornamental plants, such as *S*. *oblata*[10], *Rosa hybrida*[11], tree peony[12], *Lilium* spp.[13], and *Prunus mume*[14], have been investigated. Terpenoids have been found to be the most important components in a wide range of species[15]. Terpenoids are derived from the mevalonate (MVA) pathway in the cytosol, mainly mediating the biosynthesis of sesquiterpene, or from the plastid 2-C-methyl-d-erythritol-4-phosphate (MEP) pathway. Many of the related terpenoid biosynthetic enzymes [e.g., acetoacetyl-CoA transferase (AACT), 3-hydroxy-3-methylglutaryl-CoA synthase (HMGR), mevalonate kinase (MVK), phosphomevalonate kinase (PMK), MEP cytidylyltransferase (MCT), 4-(cytidine 5′-diphospho)-2-C-methyl-d-erythritol kinase (CMK), 2-C-methyl-d-erythritol-2,4-cyclodiphosphate synthase (MDS), 1-hydroxy-2-methyl-2-(E)-butenyl-4-diphosphate synthase (HDS), and 1-hydroxy-2-methyl-2-(E)-butenyl-4-diphosphate reductase (HDR)] have been also identified and functionally characterized[16]. However, the biosynthesis and regulatory mechanisms of floral scent, especially in woody ornamental plants, are largely unknown.

Genus *Syringa* (family Oleaceae) includes 27 wild species, most of which are distributed in China. *S*. *oblata*, a deciduous, hardy, and fast-growing perennial shrub, is native to China, and is distributed in northern regions such as Inner Mongolia, Qinghai, Hebei, Beijing, and Liaoning provinces[17]. *S*. *oblata* is widely cultivated because of its high ornamental and economic value with elegant color and unique fragrance[18]. *S*. *oblata* blooms in late April through May (depending on weather), one to two weeks earlier than its well-known lilac cousin *S*. *vulgaris* [19]. As a native and early flowering tree species in the Beijing area, *S*. *oblata* has been recommended as the foundation tree species for botanical gardens and afforestation. However the biosynthesis mechanisms of floral pigments and scent are largely unknown, especially on the molecular level, which limits the cultivation of new varieties of *S*. *oblata*.

Because of the lack of genomic evidence, the regulatory mechanisms of floral pigments and scents biosynthesis in *S*. *oblata* are difficult to investigate on the molecular level. RNA sequencing (RNA-Seq), one of the most recent Illumina sequencing techniques, has dramatically improved the efficiency of genomic exploration and gene discovery in non-model plant species for which reference genome sequence data are not available[20]. RNA-Seq generates absolute gene expression measurements and thus provides greater insight and accuracy than microarray data[20, 21]. RNA-Seq has been used to examine flower development in several garden plants, such as *Chimonanthus praecox*[22], *Cymbidium sinense*[23], *Cymbidium ensifolium*[24], and *Salvia splendens*[25], and genes related to flowering, signal transduction, and flower development have been identified. RNA-Seq provides a feasible method to investigate the metabiosynthesis and regulatory mechanisms of floral pigments and scents through transcriptomic analysis.

In this study, we used RNA-seq technology to analyze the transcriptome of *S*. *oblata* flowers in different developmental stages. Using DEGseq software [26, 27], the differential gene expression in the flower buds and in open flowers was examined. We identified genes associated with pathways of important secondary metabolic processes that were differentially expressed during flower development, which provides comprehensive information about gene expression at the transcriptional level and increase the understanding of the molecular mechanisms of flower pigment biosynthesis and floral scent metabolism in *S*. *oblata*. The data also provide an important bioinformatic resource for investigating the flowering pathway and other biological mechanisms in this and other lilac species.

## Materials and Methods

### Plant materials

**Fig 1:**
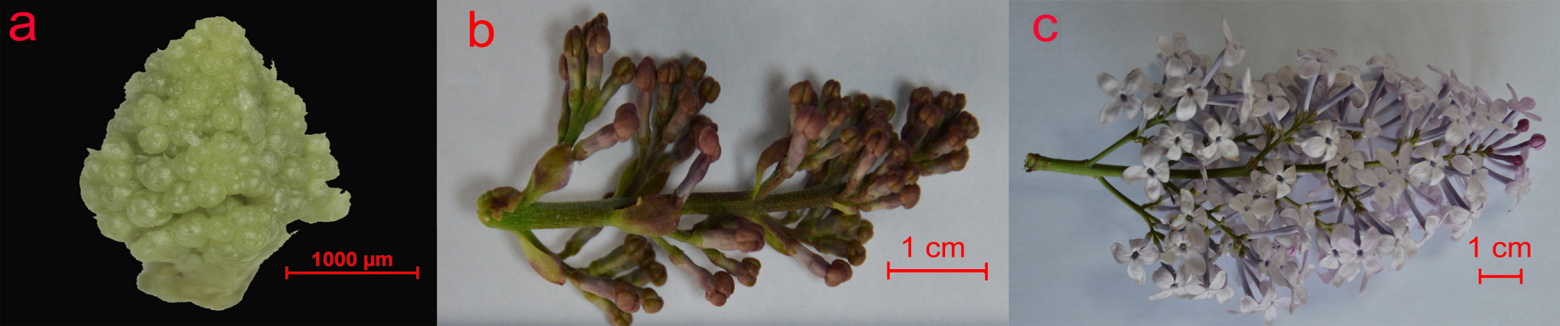
Flower development stages in *Syringa oblata*. Images of *Syringa oblata* flowers: a, at flower bud stage (SOFB); b, bud stage (SOB): c, flowering stage (SOF).

Three *S*. *oblata* plants were randomly selected from the lilac resource nursery at Beijing Agricultural University, a resource conservation unit of National Forest Genetic Resources Platform (NFGRP), Beijing, China. Three distinct stages of *S*. *oblata* flower development were defined: i) flower bud stage (SOFB), with flower buds enlarged and bud scales not protruded; ii) bud stage (SOB), with inflorescence emerged and corolla lobes not displayed; and iii) flowering stage (SOF), with flowers fully opened and emitting fragrance (Fig 1). Materials at each stage were dissected from the plants and immediately frozen and stored in liquid nitrogen prior to further analysis, with three replicates per stage.

### RNA extraction

Total RNA was extracted from the plant materials using an RNAsimple plant RNA purification kit (Tiangen Biotech, Beijing, China). The quality and quantity of purified RNA was examined using a NanoDrop ND-1000 UV/Visible spectrophotometer (Wilmington, DE) and Agilent Bioanalyzer 2100 (Agilent Technologies, Santa Clara, CA) for gel electrophoresis. For each developmental stage, equal amounts of high-quality RNA from the three plant samples were used in cDNA library construction and Illumina deep sequencing.

### Construction of cDNA library for Illumina sequencing

Nine cDNA libraries were prepared using an Illumina RNA-Seq sample preparation kit (RNeasy Micro kit, Cat.#74004, Qiagen, China) using 10 μg of total RNA. The mRNA was isolated by polyA selection with oligo (dT) beads and fragmented using fragmentation buffer. Synthesis of cDNA, end repair, A-base addition, and ligation of the Illumina-indexed adaptors were then performed according to the Illumina protocol. Libraries were size-selected on 2% Low-Range Ultra Agarose (Bio-Rad Laboratories (Shanghai) Co., Ltd) for cDNA target fragments of 300–500 bp; this was followed by PCR amplification using Phusion DNA polymerase (NEB) for 15 PCR cycles. Following quantification by TBS380, paired-end libraries were sequenced using the Illumina HiSeq 2500 system (2×100 nt multiplex) at Shanghai Biotechnology Co., Ltd. (Shanghai, China). Data analysis and base calling were performed with the Illumina instrument software.

### Sequence data analysis and *de novo* assembly

The raw reads were first filtered by removing the adapter sequences, fragments <20 bp in length, and low-quality sequences, which included reads with N percentage (the percentage of nucleotides in a read that could not be sequenced) >5% and reads containing more than 20% nucleotides with Q-value≤10. The Q-value represents the sequencing quality of related nucleotides. Using the CLC Genomics Workbench software (V6.0.4, http://www.clcbio.com/products/clc-genomics-workbench/) [28–30], clean reads from the nine libraries obtained from the three flower stages were used in *de novo* splicing and read-mapping to the transcriptome in the absence of a reference genome. The spliced sequence was called the primary unigene. The primary unigene was further assembled to the final unigene with the CAP3 EST software[31]. The final unigene was used for further analysis.

### Sequence annotation and classification

For annotation, the unigene sequences were searched against the NCBI non-redundant (NR) protein database[32] using BlastX (http://www.ncbi.nlm.nih.gov/BLAST), with a cut-off E-value of 10–5. Gene ontology (GO) terms were extracted from the annotation of high-score BLAST matches in the NCBI NR proteins database (E-value ≤ 1.0×10–5) using Blast2GO (http://www.blast2go.com), and then were sorted into categories using in-house perl scripts[33]. Functional annotation of the proteome was carried out by a BlastP homology search against the NCBI eukaryotic Orthologous Groups (KOG) database (http://www.ncbi.nlm.nih.gov/COG/). KEGG pathway annotations were performed using the online KEGG Automatic Server (KAAS, http://www.genome.jp/kegg/kaas/) [34].

### Expression analysis

Final unigenes with differential expression among the three stages of *S*. *oblata* flower development were detected with DEGseq software [26, 27]using three replicates per stage. A general Chi-squared test of statistical significance was used, and the false discovery rate (FDR) of results was controlled. If FDR was lower than 0.05 and the highest RPKM (reads per kilobase per million reads) of the unigene was twice that of the lowest one, the unigene was considered as differentially expressed.

GO enrichment analysis of differentially expressed genes (DEGs) was performed using the GOseq R package[35], which corrected for gene length bias. GO terms with corrected *P*-value less than 0.05 were considered significantly enriched by DEGs. We used the KOBAS software to test the statistical enrichment of DEGs in KEGG pathways[36].

Significantly altered genes among SOFB, SOB and SOF were described using heatmap analysis with unsupervised hierarchical clustering. The raw intensity (RPKM) was log2 transformed and then used for the calculation of Z scores[37].

### Quantitative real-time PCR (qRT-PCR) validation

Real-time PCR was performed on an ABI Power SYBR Green PCR Master Mix and 7900 HT Sequence Detection System (Applied Biosystems). Sequences of the specific primer sets are listed in the S1 File. The ubiquitin domain-containing protein gene (*DSK2B*, Acc. No.Q9SII8) and actin gene (*Actin-7*, Acc. No. P53492) of *S*. *oblata* were used as internal markers. qRT-PCR was performed using the SYBR Premix Ex Taq Kit (TaKaRa) according to the manufacturer’s protocol. The results were normalized to the expression level of the constitutive ubiquitin and actin genes. A comparative Ct method (2^–△△Ct^) of relative quantification was used to evaluate the quantitative variation. All quantitative PCR for each gene used three biological replicates, with three technical replicates per experiment. The RNA samples for qRT-PCR were the same as those for Illumina sequencing.

## Results

### Illumina sequencing and assembly of sequence reads

**Fig 2:**
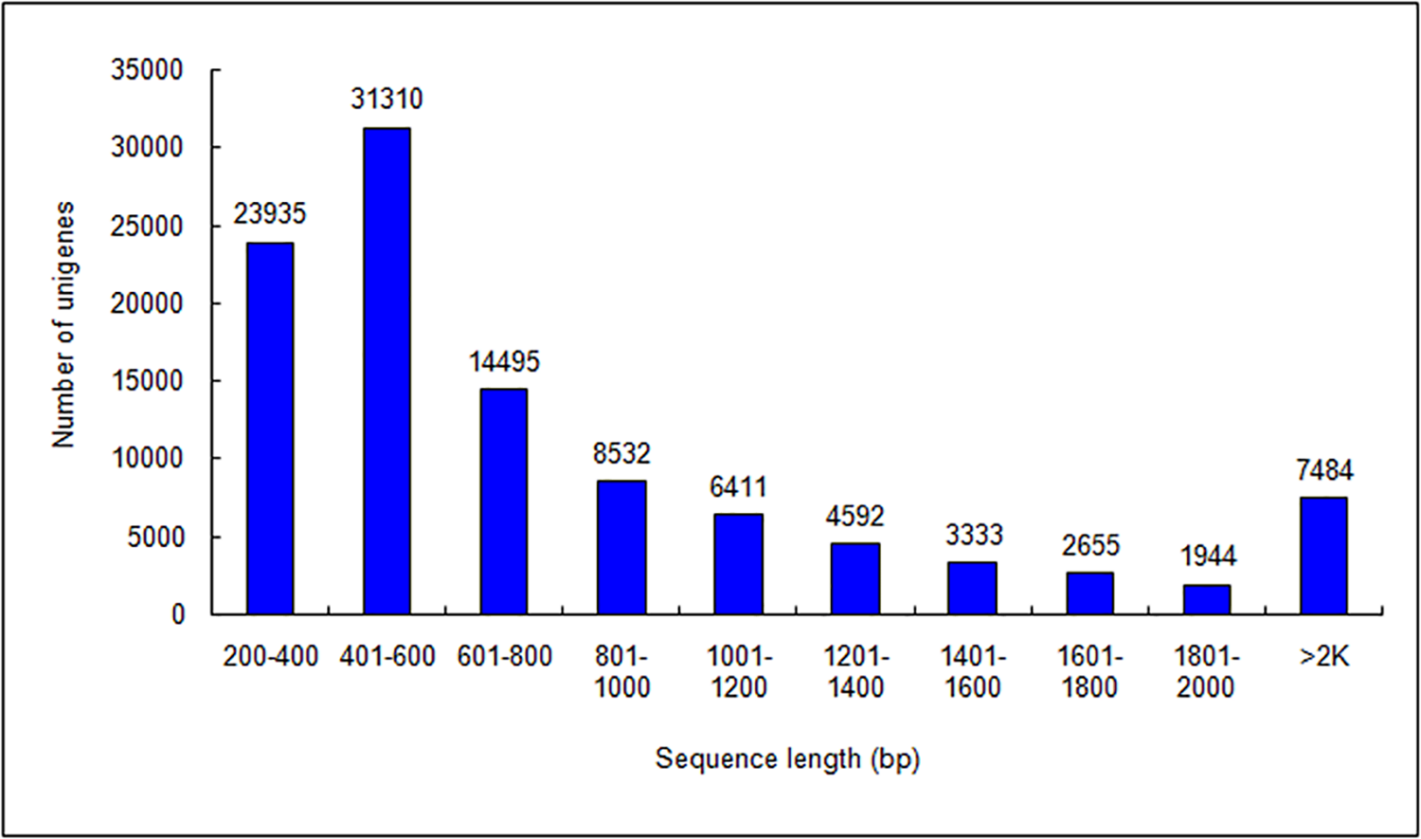
Length distribution of assembled *Syringa oblata* unigenes. All clean reads for each flower development stage (see Fig 1) were combined and resulted in 104,691 unigenes. Horizontal and vertical axes show the size and the number of unigenes, respectively.

In this study, nine cDNA libraries were constructed and subjected to Illumina deep sequencing. We obtained 337,669,638 raw reads and 319,425,972 clean reads from the three developmental-stage libraries after eliminating primer and adapter sequences and filtering out low-quality reads. We obtained 130,981 contigs from these reads. Based on the paired-end reads, these contigs were further assembled into primary unigenes using CLC Genomics Workbench (version: 6.0.4; Table 1). Finally, using CAP3 EST, we spliced the primary unigenes a second time and obtained a transcriptome database for *S*. *oblata*. The *de novo* assembly generated 104,691 final unigenes (Table 1). The distribution of unigene sequence length is shown in Fig 2. Approximately 30% of the unigenes were 401–600 bp in length, 23% were 201–400 bp, and 47% were >600 bp (Fig 2).

**Table 1:** Summary of assembly statistics for *Syringa oblata* transcriptome.

Statistics	Counts	Total length (bp)	N50 (bp)	Average length (bp)	Longest (bp)
Contigs	130,981	89,118,182	858	680	15,223
Primary unigenes	106,708	89,735,247	1,077	841	15,756
Final unigenes	104,691	89,306,170	1,099	853	15,756

### Unigene annotation

**Fig 3:**
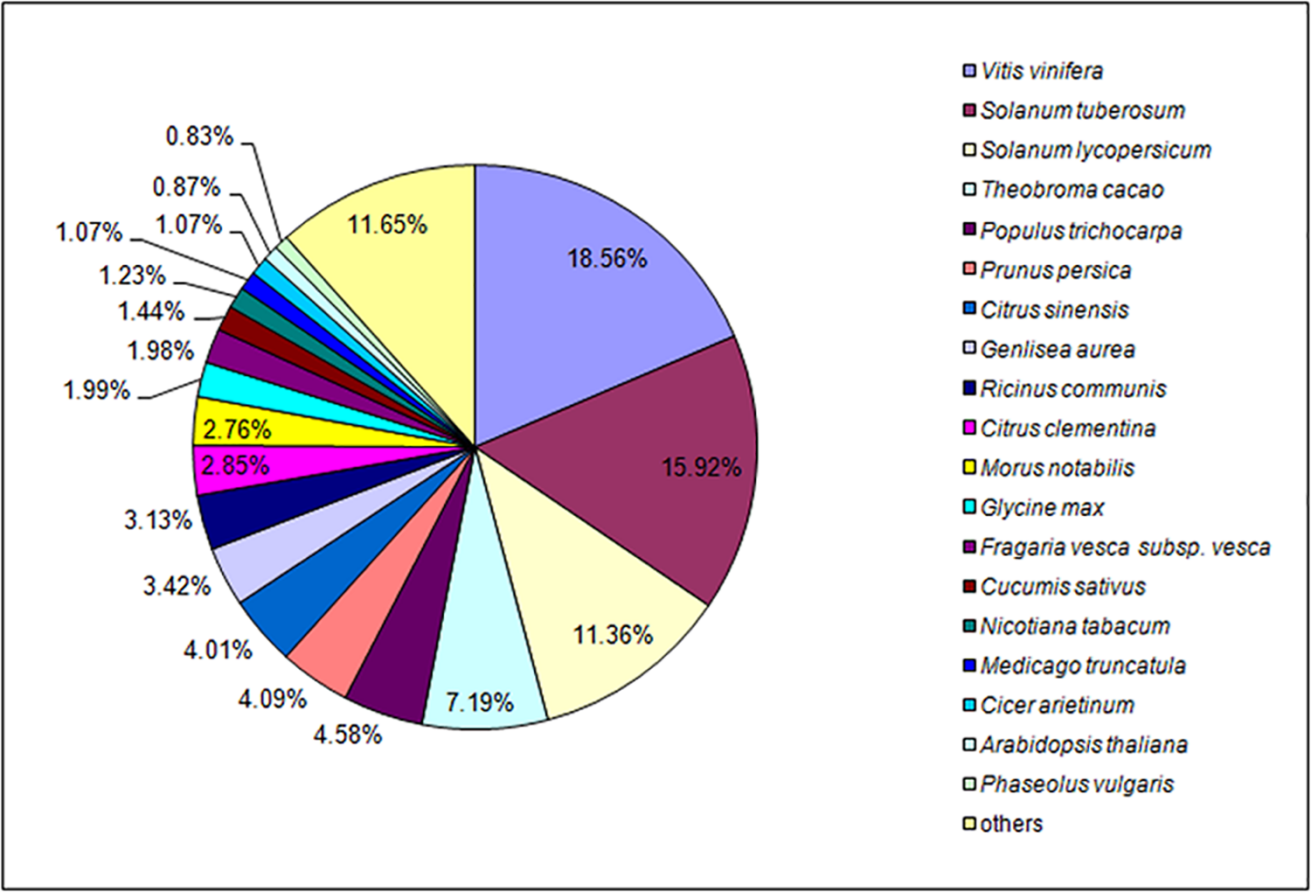
Species-based distribution of BLASTX matches for unigenes against NCBI NR database. We used all plant proteins in the NCBI NR database in performing the homology search; for each sequence, we selected the closest match for analysis.

All unigenes for *S*. *oblata* were searched against the NCBI non-redundant (NR) database using BlastX with a cut-off E-value of 10–5. Among these unigenes, approximately 42% showed significant similarity to known proteins in the NR database, and 29% were matched to the SwissProt database (Table 2). In further analysis of the matching unigenes, we found that *S*. *oblata* unigenes were most closely matched with gene sequences from *Vitis vinifera*, followed by *Solanum tuberosum*, *S*. *lycopersicum*, *Theobroma cacao*, and *Populus trichocarpa* (Fig 3).

**Table 2:** Statistics of annotation results for *Syringa oblata* unigenes.

Database	NR	SwissProt	GO	KOG	KEGG	All
Number annotated	43,712	30,262	36,967	19,956	12,388	44,164

In addition to the NCBI NR and SwissProt databases, *S*. *oblata* unigenes were aligned with Gene Ontology (GO), eukaryoticorthologous groups of proteins (KOG), and the Kyoto Encyclopedia of Genes and Genomes (KEGG). In total, 42% of the unigenes provided significant BLAST results; of these, approximately 84% had matches in the GO database, 45% had matches in the KOG database, and 25% had matches in KEGG (Table 2). The significance of a BLAST search depends on the length of the query sequence; thus, short reads obtained from sequencing are rarely matched to known genes [38]. Approximately 23% of the unigenes we identified were short (200–400 bp), which might help to explain the proportion that did not match. In other words, these short reads generated by sequencing or assembly might have resulted in the low significance[25].

### GO classification of unigenes

**Fig 4:**
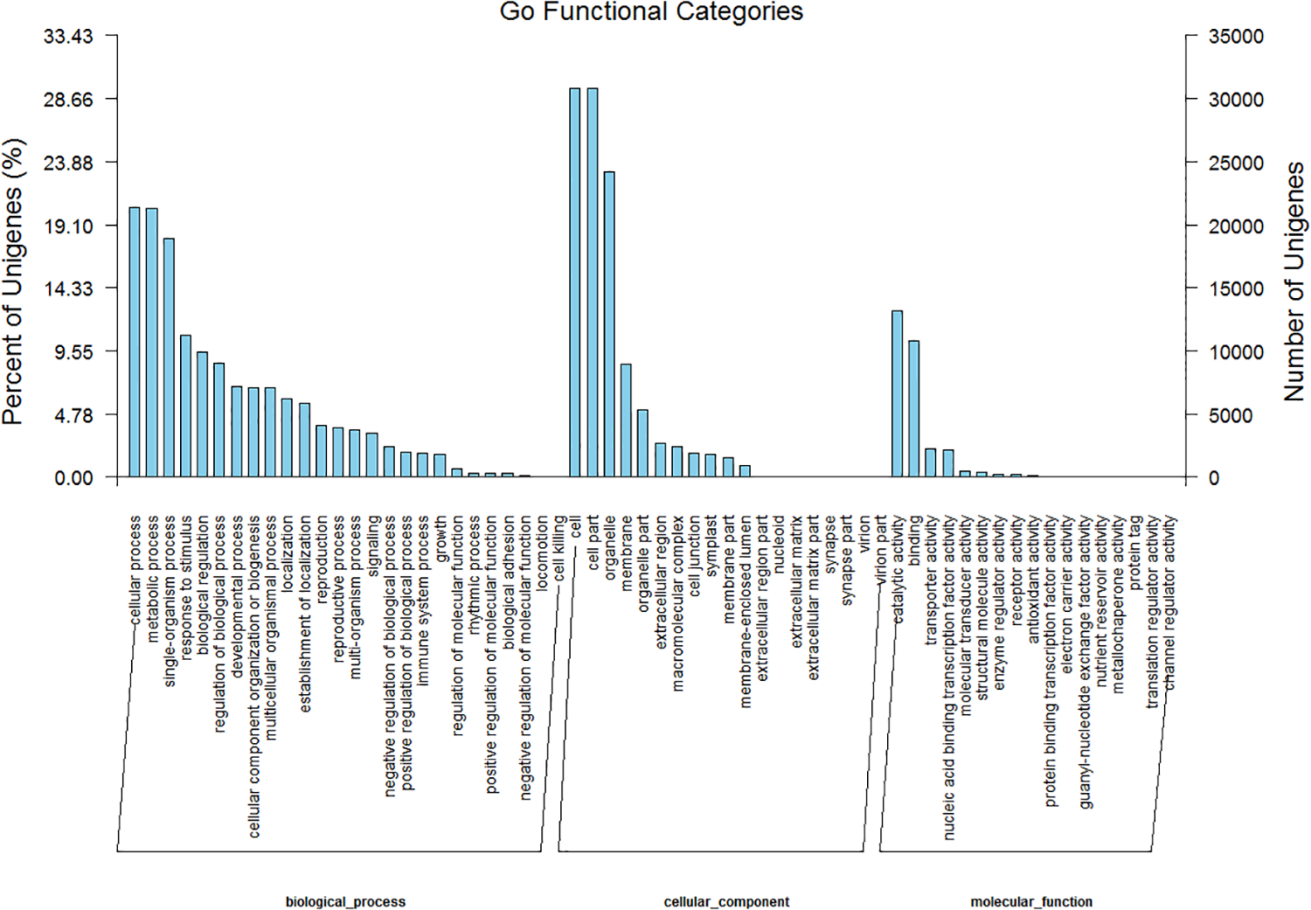
Gene ontology (GO) classification of unigenes of *Syringa oblata* inflorescence. Left y-axis indicates the percentage of unigenes in subcategories of each main category. Right y-axis indicates the number of unigenes in each subcategory.

We used Blast2GO to classify the functions of the predicted *S*. *oblata* unigenes. Of the 104,691 final unigenes, 36,967 were successfully annotated by GO assignments. The annotated unigenes were assigned into three main GO categories and 62 subcategories, and some belonged to more than one of the three categories (Fig 4). Based on annotation against the NR database, 33,208 GO terms were assigned. The greatest proportion (~62%) was assigned to biological processes (GO: 0008150); the others were assigned to molecular functions (GO: 0005575; ~24%) or cellular components (GO: 0003674; ~14%) (Fig 4).

Among the biological processes, dominant subcategories included cellular, metabolic, and single-organism processes and response to stimulus and biological regulation were also important (Fig 4). The large proportion of annotated unigenes involved in metabolic processes suggested that novel genes involved in pathways of secondary metabolite synthesis could be identified. Dominant subcategories of cellular components included cells, cell parts, organelles, and membranes. Molecular function subcategories with the largest numbers of annotated unigenes were catalytic activity, binding, transporter activity, and nucleic acid binding transcription factor activity (Fig 4, S2 File).

### KOG classification of unigenes

**Fig 5:**
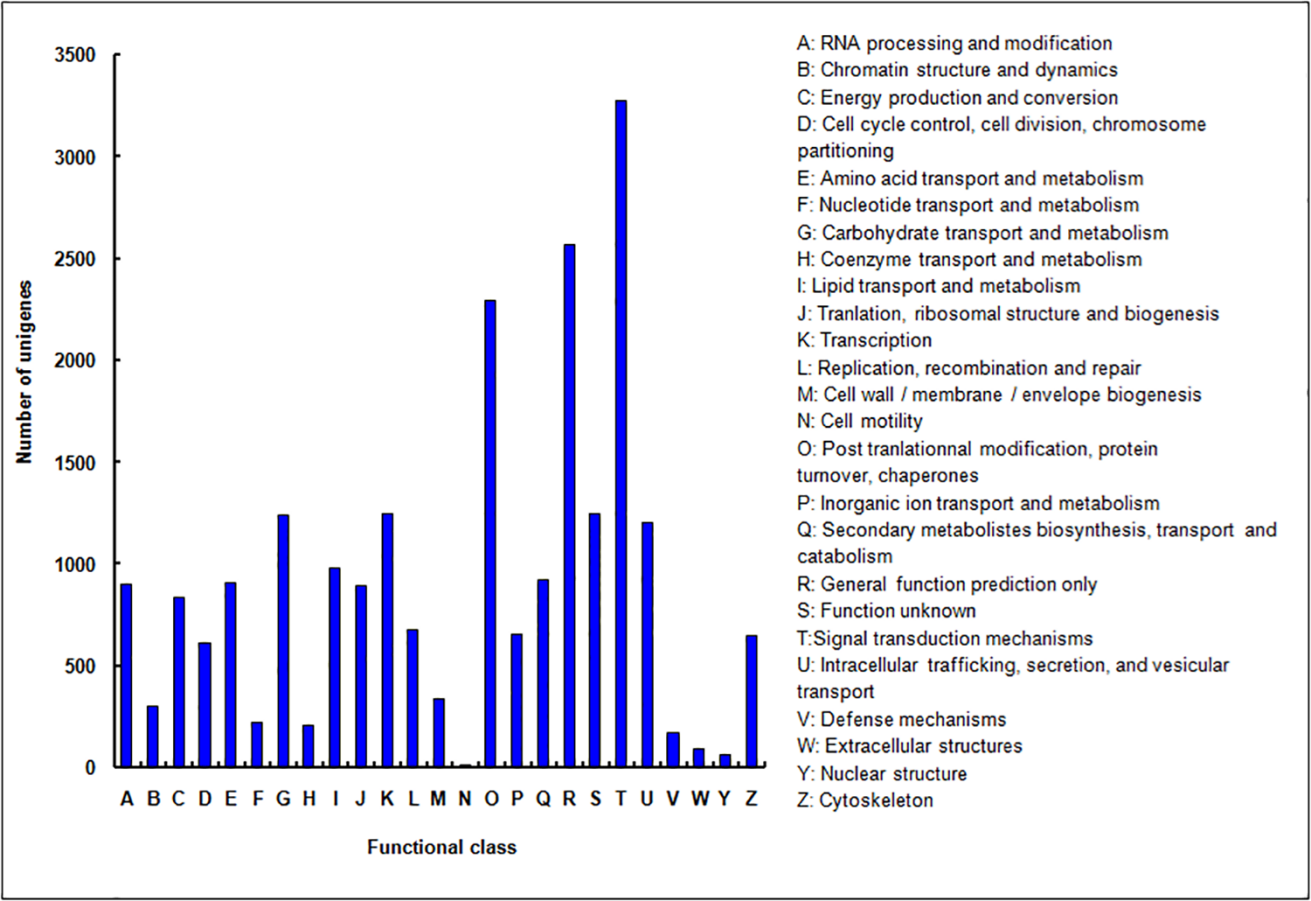
KOG functional classification of unigenes of *Syringa oblata* inflorescence. From a total of 104,691 final unigenes, 19,956 annotated unigenes with significant homology in the KOG database (E-value ≤1.0 E^–^5) were classified into 25 KOG categories.

The KOG database is a useful platform for functional annotation of newly sequenced genomes. Based on sequence homology, 19,956 unigenes were assigned to the KOG functional classification into 25 categories denoting their involvement in cellular processes, biochemistry and metabolism, signal transduction, and other functions (Fig 5). Signal transduction mechanisms were dominant, and general functional prediction and posttranslational modification–protein turnover–chaperones shared a large proportion of genes (Fig 5, S3 File). However, 924 unigenes (4.6%) were assigned to the category of secondary metabolite biosynthesis, transport, and catabolism, which suggests that identification of novel genes involved in secondary metabolism pathways is a promising area of study (S3 File).

### Metabolic pathway assignment by KEGG

The KEGG database places emphasis on biochemical pathways and provides an alternative approach to the categorization of gene function. Here, 12,388 annotated unigenes were assigned to 286 KEGG pathways (S4 File). Metabolic pathways had the most representation (~23%), followed by biosynthesis of secondary metabolites (~13%). Several signaling pathways (AMPK, MAPK, p53) and biosynthesis pathways of important secondary metabolites (e.g. phenylpropanoid, carotenoid, flavonoid, flavone and flavonol, anthocyanin, volatile terpenoids) were represented (S4 File). These data provide a valuable resource for further mining pathways of interest in *S*. *oblata*.

We focused on the metabolic pathways involved in flower pigments and scents biosynthesis. Flavonoids are primary compounds affecting the formation of flower or fruit color, and three pathways are involved in flavonoid metabolism: the anthocyanin, flavonoid, and flavone/flavonol biosynthetic pathways [39]. Among these pathways, 55 unigenes (average length,1130 bp) were associated with flower pigment synthesis, including 16 unigenes annotated to anthocyanin biosynthesis, 31 unigenes annotated to flavonoid biosynthesis, and 14 unigenes annotated to the flavone/flavonol biosynthetic pathway (S5 File). Further analysis revealed that nine unigenes were between 300 and 500 bp, 20 unigenes were between 500 and 1000 bp, and 26 unigenes were >1000 bp; moreover, all unigenes had very high homology with the NR database (E <10^–12^) (S5 File). In addition, six unigenes were annotated to both the flavonoid and the flavone/flavonol biosynthetic pathway, which suggested that these genes could be key nodes in the three flavonoid metabolic pathways (S5 File).

Volatile terpenoids are important components of the floral scent in *S*. *oblata*[40]. Three metabolic pathways: terpenoid backbone biosynthesis, monoterpenoid biosynthesis, and limonene and pinene degradation contributed to the biosynthesis of terpenoid volatiles, and 213 unigenes (average length,1069 bp) were annotated (S5 File). Among these, 45% of the unigenes were involved in limonene and pinene degradation, 43% were involved in terpenoid backbone biosynthesis, and only 25 unigenes were clustered in monoterpenoid biosynthesis (S5 File).

This analysis of the metabolic pathways of flower pigment and floral scent helps to provide a basis for cloning and functional analysis of the key genes involved these important characteristics of *S*. *oblata*.

### Variation in gene expression among flower development stages

**Fig 6:**
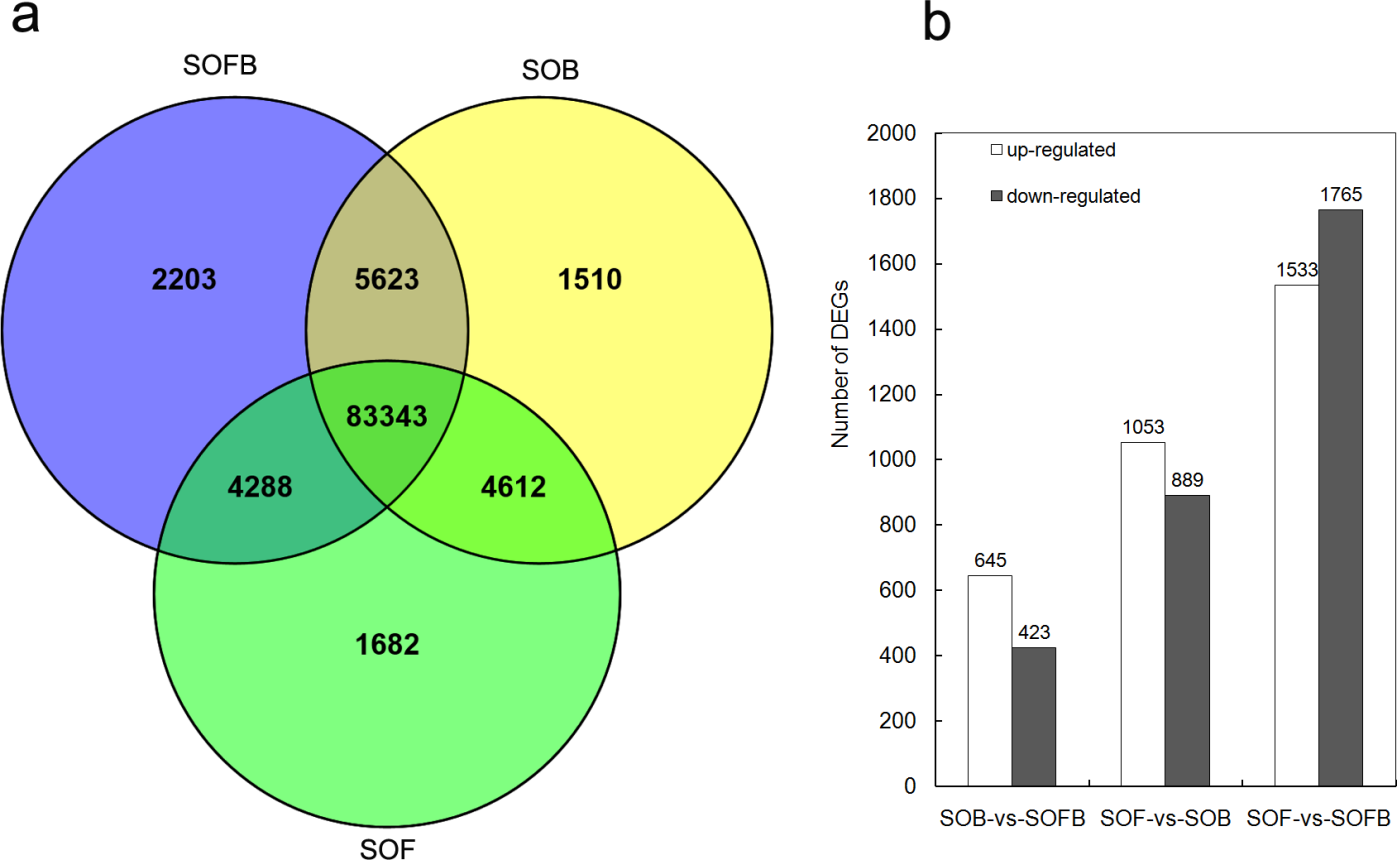
Distribution of genes among each flower developmental stage. a: Venn diagram illustrating the expression patterns of genes among flower developmental stages. b: Number of DEGs in each flower developmental stage. DEGs are compared between developmental stages. SOFB, flower bud stage; SOB, bud stage; SOF, flowering stage.

Among the annotated unigenes, we found that 83,343 genes were expressed in samples of all three developmental stages, and 2,203, 1,510, and 1,682 genes were found to be expressed specifically in the SOFB, SOB, and SOF stages, respectively (Fig 6A). To identify differentially expressed genes (DEGs) during flower development, we performed genome-wide expression analysis for the SOFB, SOB, and SOF developmental stages (Fig 1). Following Anders and Huber [27], we identified genes that were differentially expressed between two samples by comparing SOB and SOFB, SOF and SOB, and SOF and SOFB. In total, 1,068 differentially regulated genes, including 645 upregulated and 423 downregulated genes, were identified in SOB compared to SOFB (Fig 6B). The functional annotations and statistics for all DEGs between SOB and SOFB are in the S6 File. We also classified the functions of the 1,068 DEGs by Blast2GO. Among biological processes, dominant subcategories included metabolic processes, cellular processes, and single-organism processes. In cellular components, the two largest subcategories were cell and cell parts. Catalytic activity was dominant in the molecular functions section (S7 File). GO enrichment analysis showed that most of the upregulated DEG sets were related to biological processes, cellular components, molecules, and DNA binding (S8 File). The KEGG enrichment classification showed that 23 groups were significantly enriched, and most of these were upregulated primarily in the SOB library, which was correlated with metabolic and biosynthesis pathways of secondary metabolites (S8 File). Based on sequence homology, the 1,068 DEGs were assigned to the KOG functional classification into 23 categories (S1 Fig).

Compared with SOB, more DEGs were identified in SOF (Fig 6B), and the statistical information for these DEGs is summarized in the S6 File. The results of the GO functional classification between the SOF and SOB stages were similar to those for the comparison of SOB to SOFB (S9 File). GO enrichment analysis indicated that approximately half of the gene sets were downregulated and half were upregulated in the SOF library. The downregulated genes were mainly related to responses, chloroplasts, membranes, and cytosol, and the upregulated genes were related to molecular functions, kinase activity, nuclei, and biological processes (S10 File). In the KEGG classification, 32 groups were significantly enriched, and about half of these genes were upregulated and half were downregulated in the SOF library. These enriched genes were mainly related to metabolic pathways and biosynthesis of secondary metabolites (S10 File). The KOG functional classification showed that the DEGs between the two stages were also assigned to 23 categories (S2 Fig).

We also compared gene expression between SOF and SOFB, and found 3298 genes with differential expression (Fig 6B). The functional annotations and statistical information of all 3298 DEGs are summarized in the S6 File. GO functional classification indicated that the most of the 3298 DEGs were involved in metabolic processes, cellular processes, and single-organism processes in the biological process section; the dominant subcategories were cell and cell parts in the cellular components section; and catalytic activity was the dominant subcategory in the molecular functions section (S11 File). GO enrichment analysis showed that most of the gene sets were downregulated in the SOF library, and these genes were mainly related to epigenetics, flower development, cytosols, and binding (S12 File). KEGG classification showed that 14 groups were significantly enriched, and most of these genes were downregulated in the SOF library and were related to ribosomes and DNA replication (S12 File). In KOG functional classification, the DEGs between the two stages were also assigned to 24 categories (S3 Fig).

### Genes related to flower pigment biosynthesis and floral scent metabolism

**Fig 7:**
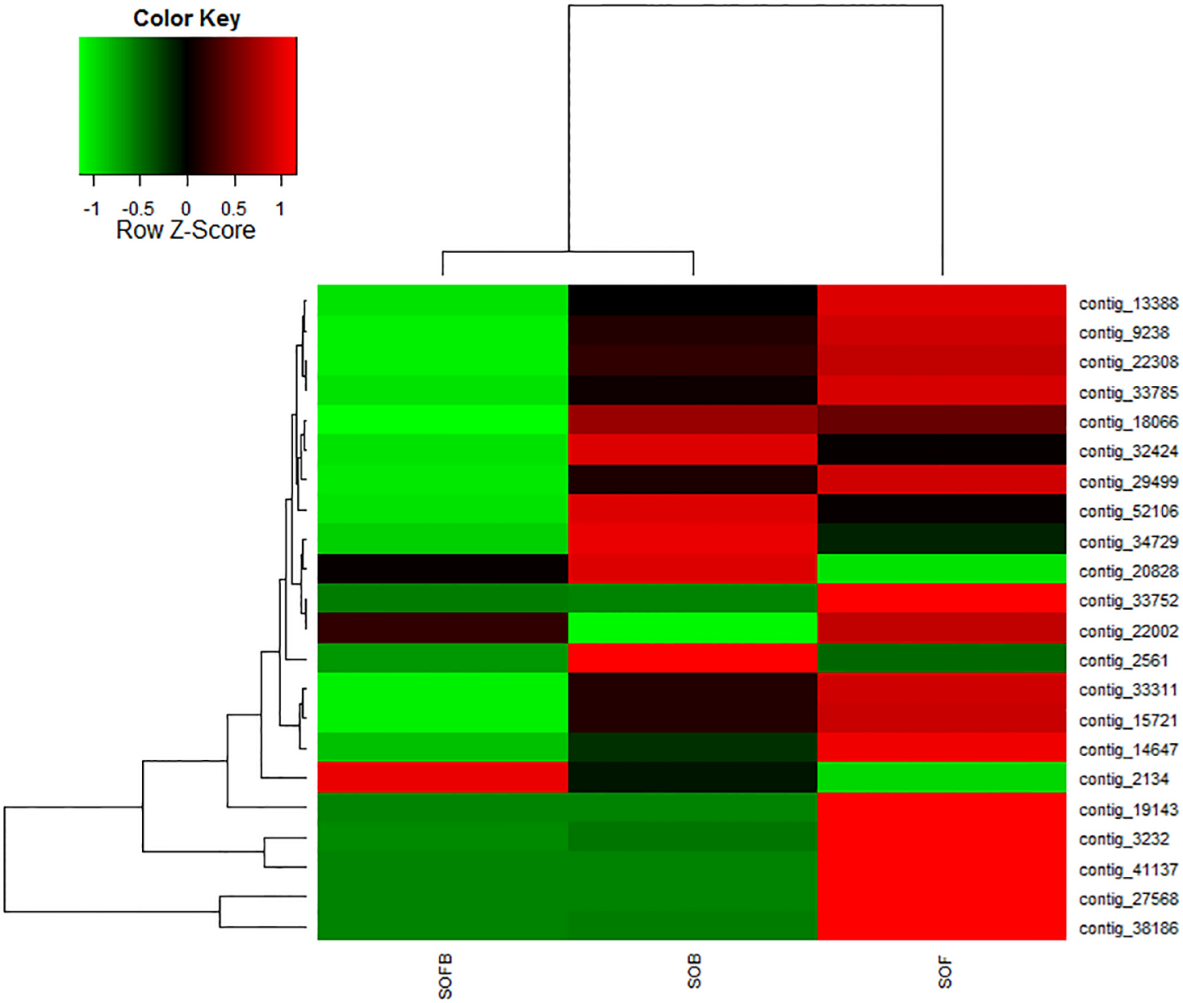
Differential expression of genes related to flower pigmentation and floral scent biosynthesis in *Syringa oblata*. Each column represents an experimental sample (SOFB, SOB, and SOF), and each row presents a gene. Expression differences are shown by different colors. Red indicates high expression and green indicates low expression.

During development, the flowers of *S*. *oblata* underwent rapid changes in color and scent, from green and unscented (SOFB) to amaranth and slightly scented (SOB), and then to lilac with very strong scent (SOF). We focused on genes related to flower pigment biosynthesis and scent metabolism, and chose some genes that showed significant differential expression among the three developmental stages (Fig 7). The functional annotation for these unigenes was provided in S13 File. For example, contig_2561 showed lower expression levels in SOFB and SOF than in SOB and was homologous with *Catharathus roseus* cinnamic acid 4-hydrolase (C4H), related to flavonoid biosynthesis. Other genes associated with anthocyanins biosynthesis showed higher expression levels in SOB and SOF than in SOFB. These genes included contigs_52106 (homologous with *Perilla frutescens* chalcone synthase, CHS), 14647 (homologous with *Camellia chekiangoleosa* flavanone 3-hydroxylase, F3H), 9238 (homologous with *Petunia×hybrida* flavonol synthase, FLS), 22308 (homologous with *Eustoma grandiflorum* anthocyanidin synthase, ANS), and 32424 (homologous with the *Olea europaea* flavonoid 3-O-glucosyltransferase, 3GT). In addition, some genes of anthocyanin biosynthesis pathways, such as contig_38186 (homologous with COMT1), contig_27568 (homologous with DOMT-like) and contig_33752 (homologous with GT) displayed the highest expression in the SOF stage, which indicated that these genes located downstream of anthocyanin biosynthesis. In particular, the mean FPKMs of contig_27568 were 1.08, 1.43, and 4295 in each SOFB, SOB, and SOF stage, respectively (P = 0), which means that the *DOMT-like* gene might be specifically and strongly expressed at the SOF stage (S6 File).

Some key genes related to fragrance biosynthesis showed a significant difference in expression among the three flower developmental stages. The functional annotation for these unigenes is shown in the S13 File. The contigs_3232 (homologous with *Salvia miltiorrhiza*1-deoxy-d-xylulose 5-phosphate synthase, DXS), 33311 (homologous with *Nicotiana tabacum* hydroxy-methylglutaryl-coenzyme A reductase, HMGR), 13388 (homologous with *S*. *miltiorrhiza* geranyldiphosphatesynthase, GPS), and 33785 (homologous with *Oryza sativa* subsp. *Japonica*1-deoxy-d-xylulose 5-phosphate reductoisomerase, DXR) had the highest levels of expression in SOF and encoded key enzymes involved in biosynthesis of terpenoid volatiles; however, the three terpenoid synthase genes with contigs_22002 (homologous with camphene/tricyclene synthase [TPS3] of *S*. *lycopersicum*), 19143 (terpene synthase[TPS] of *O*. *europaea*), and 41137 ([3S]-linalool/[E]-nerolidol synthase [LIS] of *V*. *vinifera*) showed the highest expression levels in SOF. In contrast, contigs_2134 and 18066 from terpene degradation genes showed the highest expression in SOFB and SOB, respectively. In addition, we found that some cytochrome P450 genes involved in metabolism of terpenoid volatiles, such as contigs_2134 (homologous with *V*. *vinifera* cytochrome P450 76A2, CYP76A2), 819 (homologous with *A*. *thaliana* cytochrome P450 78A7, CYP78A7), and 17171 (homologous with *V*. *vinifera* cytochrome P450 77A3, CYP77A3), showed the highest expression in SOFB stage. However, contig_18066 (homologous with *V*. *vinifera* cytochrome P450 77A2, CYP77A2) displayed higher expression in SOB and SOF than in the SOFB stage. Finally, we found that three genes, *DXS* (contig_3232), *TPS* (contig_19143), and *LIS* (contig_41137), were induced specifically and strongly (>100-fold change in expression) at the SOF stage (S6 File).

Apart from the structural genes, nine transcription factors regulating anthocyanin and fragrance biosynthesis were found in this study (S6 File). We found that the six genes encoding the transcription factors [e.g., contigs 28146 (homologous with *Prunus avium* transcription factor R2R3-MYB), 28147 (homologous with *Theobroma cacao* transcription factor MYB), 38962 (homologous with *Camellia sinensis* transcription factor R2R3-MYB1), 25616 (homologous with *Citrus sinensis* transcription factor bHLH79), 9153 (homologous with *S*. *tuberosum* transcription factor bHLH147), and 64997 (homologous with *Fragaria vesca* bHLH79-like transcription factor)] showed higher expression in SOB and SOF than in the SOFB stage. However, the other three genes [e.g., contigs_11688 (homologous with *A*. *thaliana* transcription factors MYB44), 21735 (homologous with *S*. *tuberosum* transcription factor bHLH3), and 20570 (homologous with *A*. *thaliana* transcription factor bHLH48)] displayed higher expression in SOFB than in the other two stages (S6 File).

### Real-time Quantitative PCR Validation of RNA-seq Results

**Fig 8:**
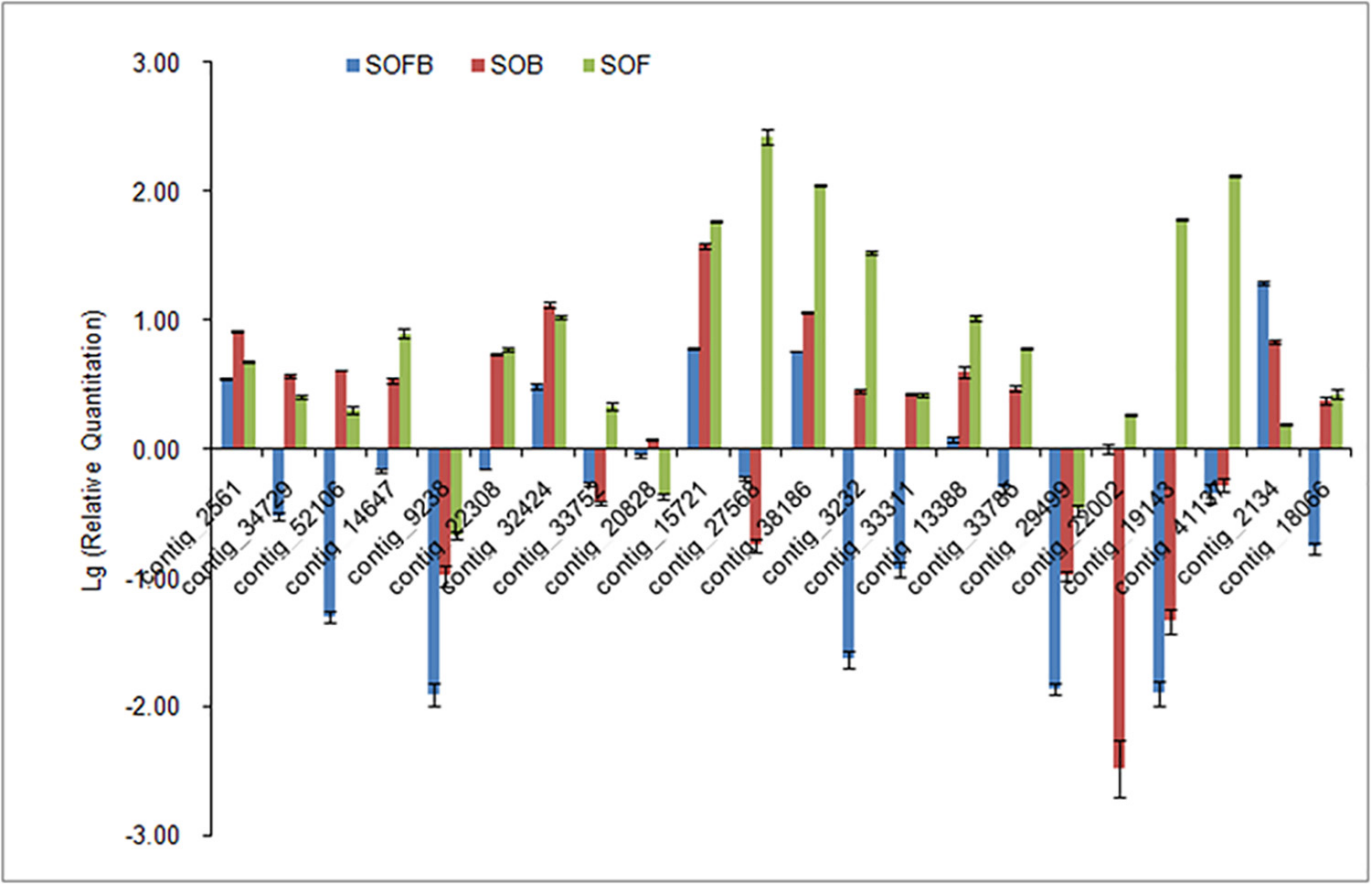
Real-time qPCR validation of genes involved in flower pigmentation and scent biosynthesis in *Syringa oblata*. The y-axis indicates fold changes in expression among flowering stages (SOFB, SOB, and SOF) using results from RT-qPCR. Data were normalized against *Syringa oblata* reference gene *DSK2B*. Quantitative PCR for each gene used three biological replicates, with three technical replicates per experiment. Bars indicate SD.

To validate the sequencing data, 22 genes involved in flower pigment biosynthesis and fragrance metabolism were selected for real-time qPCR with gene-specific primers designed using Primer version 5.0 (S1 File). The expression patterns of these genes in each flower developmental stage are shown in Fig 8. Using *S*. *oblata DSK2B* as a reference gene, expression patterns determined by real-time qPCR were consistent with those obtained by RNA-Seq (Fig 7, Fig 8), confirming the accuracy of the RNA-Seq results. We also obtained identical real-time qPCR results using *S*. *oblata ACT-7* as a reference gene (S4 Fig).Thus, the data generated here can be used to investigate specific flowering genes that show comparative expression levels among different developmental phases.

## Discussion

### Illumina sequencing and sequence annotation

*S*. *oblata* is a popular and traditional garden plant in many countries, including China. However, little is known about the mechanisms responsible for floral color and fragrance, and genomic information for this species is currently unavailable. The aims of this project were to generate a large amount of cDNA sequence data that would facilitate more detailed studies of *S*. *oblata* and to identify genes that control flower pigments and floral scents compounds. The availability of transcriptomic data for *S*. *oblata* will meet the initial informational needs for functional studies (e.g., molecular genetics, variety breeding, biochemical characterization) of this species and its relatives. Here, RNA-seq was performed using Illumina sequencing and generated 319,425,972 high-quality reads that were assembled into 104,691 final unigenes with an average sequence length of 853 base pairs. The average length of the assembled unigenes was greater than that obtained for *C*. *sinense* (612 bp)[23], *Myrica rubra* (437 bp)[41], bamboo (736 bp)[42], and *Hevea brasiliensis* (485 bp)[43] using similar sequencing technologies. For gene annotation, the 104,691 final unigenes were used for BLASTX and annotation against NCBI protein databases including NR, SwissProt, KOG, KEGG, and GO. Because of the limited availability of genetic information, 44,164 unigenes were identified through BLAST searches, and 57.81% of unigenes had no homologues in the NCBI databases. Interestingly, similar to the results for *C*. *praecox*[22], the annotated unigenes of *S*. *oblata* showed higher homology to those of *V*. *vinifera* (Fig 3), which may reflect a closer evolutionary relationship between the two species.

### Anthocyanin biosynthesis genes in *S*. *oblata*

A set of genes that includes *CHS*, *CHI*, *F3H*, *F3*′*H*, *F3*′,*5*′*H*, *DFR*, *ANS*, *GT*, *AT*, and *MT* affect anthocyanin biosynthesis[44–50], and these genes are divided into upstream and downstream structural genes. Most of the upstream genes belong to the family of early biosynthesis genes (*EBGs*), and the downstream genes belong to the family of late biosynthesis genes (*LBGs*)[51, 52]. All genes in the anthocyanin biosynthesis pathway showed differential expression patterns during plant growth and development, consistent with findings in other species. In *Lilium* ‘Asiatic lightning’, the expression of *CHS* and *DFR* increases with flower development and reaches a maximum in the flowering period, which suggests that the gene expression is coordinated with the production of anthocyanins[53]. Three patterns of gene expression are observed in *Gentiana triflora*: *CHS* and *CHI* are expressed throughout flower development, *F3*′*H* is highly expressed in the early stage of flower development, and *F3H*, *F3′5′H*, and *DFR* are expressed only in the late stage of flower development[54]. In *Pericallis hybrida*,*CHS*, *CHI*, *F3H*, *F3*′*H*, *DFR*, *F3*′*5*′*H*, and *3MaT* are expressed at high levels in early stages, then the expression decreases moderately until there is low expression of these genes in petals in the late flowering stage[55]. In this study, we found that the upstream genes, *C4H* and *CHS*, and the downstream genes, *3GT/UDGT*, *F3H*, *FLS*, *ANS*, *coAOMT*, *DOMT-like*, and *COMT-1*, were upregulated in SOF relative to SOFB. *C4H*, *CHS*, and *3GT/UDGT* were expressed at a high level in the SOB stage (Fig 7, Fig 8). Interestingly, the *MTs* (including *coAOMTs*, *DOMT-like*, *COMT-1*) were expressed at a low level in the SOFB stage and at the highest level in the SOF stage, which indicated that the production of pigments in *S*. *oblata* was coordinated with the methylation of anthocyanidins derived from leucoanthocyanidins catalyzed by ANS. These results showed that the expression patterns of genes related to anthocyanin biosynthesis in *S*. *oblata* were similar to those of *Lilium* ‘Asiatic lightning,’ *G*. *triflora*, and *P*. *hybrida*, but were yet unique.

### Volatile terpenoid metabolism genes in *S*. *oblata*

The biosynthesis and emission of terpenes have been investigated in many plants, including snapdragon[56, 57], *Clarkia breweri*[58], *A*. *thaliana*[59], and *Lavandula angustifolia*[60]. In *S*. *oblata*, terpenoid biosynthesis genes involved in the MVA and MEP pathways were identified as *DXS*, *DXR*, *HMGR*, *GPS*, *TPS3*, *TPS*, and *LIS*. These genes showed similar expression patterns across the development stages and had the strongest expression during the SOF stage, which may result in the emission of volatile terpenoids in a large quantity, which we found in the full flowering stage (in press). The *DXS*, *DXR*, and *LIS* genes isolated from *R*. *rugosa* flowers show consistent expression during development, from budding to the withering stage[61]. In *L*. *angustifolia*, the expression of two *TPS* genes is positively correlated with the emission of volatile terpenoids during development of the inflorescence [60]. In snapdragon flowers, transcript levels of genes including *DXS*, *DXR*, *MCT*, *CMK*, and *TPS* in the MEP pathway (leading to formation of volatile terpenoids) are upregulated during petal development, implying that transcriptional induction of the MEP pathway precedes scent formation[57]. However, the expression levels of genes in the MVA pathway are not significantly upregulated with petal development, with the exception of *TPS* downstream[57]. The MEP and MVA pathways were both activated with flower development in *S*. *oblata*, in contrast to the findings in snapdragon. Thus, the distinct expression patterns of MEP and MVA pathway genes of different plants might result in differences in biosynthesis and emission of floral terpenoids. Interestingly, when the MEP and MVA pathways were stimulated, genes for terpene-degrading enzymes (contigs 2134 and 18066) showed lower expression levels, thus maintaining the release of strong floral scent during blooming (Fig 7, Fig 8).

Cytochrome P450s are important in the oxidative, peroxidative, and reductive metabolism of numerous and diverse endogenous compounds including terpenoids. In this study, we found one cytochrome P450 gene (*CYP77A2*) that was up-regulated significantly at the SOB stage and down-regulated at the SOF stage (S6 File), which was similar to a previous report of expression of a cytochrome P450 gene in winter sweet[22]. However, the other cytochrome P450 genes (*CYP76A2*, *CYP78A7*, and *CYP77A3*) were down-regulated at the SOB and SOF stage in *S*. *oblata*. Cytochrome P450 genes are found in all kingdoms and show extraordinary diversity in their chemical reactions. Although cytochrome P450 genes are one of the largest gene families in plants, their functions in flowers are as yet largely unknown.

### Transcriptional regulation of anthocyanin biosynthesis in *S*. *oblata*

Transcription factors (e.g., R2R3-MYB, bHLH, and WD40 proteins) could activate or inhibit expression of structural genes to regulate the biosynthesis of anthocyanins. Previous studies have shown that anthocyanin biosynthesis depends on regulation of MYB-bHLH compounds in some plants, e.g., in *G*. *hybrida*, interaction of GhMYB10 (R2R3-MYB) and GhbHLH encoded by *GhMYC1* activates the expression of *GhDFR* [7]. However, GtbHLH1 interacting with GtMYB3 jointly promote the expression of *GtF3'5'H* and *Gt5AT*, but were found not to influence the activity of *GtCHS* in *G*. *triflora*[8]. LhbHLH2 was shown to interact with LhMYB6 and LhMYB12, which activate the expression of *LhDFR*, *LhCHSa* and *LhCHSb* in *Lilium* spp. [9]. In addition, anthocyanin biosynthesis is also regulated by MYB-bHLH-WD40 compounds (MBW compounds) in some plants, e.g., in *A*. *thaliana*, anthocyanin biosynthesis in seeds and vegetative organs is regulated by MBW compounds such as TT2 (R2R3-MYB protein)-TT8 (bHLH042)-TTG1 (WD40 protein) compounds that promote the biosynthesis of procyanidins in seeds [6]. In petunias, the structural genes, e.g., LBGs (*DFR*, *ANS13*, *GT*, *AOMT*) and *CHSJ*, for anthocyanin biosynthesis are also regulated by MBW compounds; however, the EBGs (*CHSA*, *CHI*, *F3H*) cannot be regulated by MBW compounds[5]. In this study, we found that three MYB (R2R3-MYB, MYB, and R2R3-MYB1) and three bHLH (bHLH79, bHLH147, and bHLH79-like) transcription factors were up-regulated at the SOB and SOF stages, and another three transcription factors (MYB44, bHLH3, and bHLH48) were down-regulated at the SOB and SOF stages in *S*. *oblata* (S6 File). Yet, the molecular regulation mechanism for flower pigment biosynthesis depending on MYB-bHLH or MBW compounds still needs further research. MYB and bHLH transcription factors involved in anthocyanin biosynthesis and their roles in the regulation of structural gene expression have been studied in model and some ornamental plants, which could provide a good reference for relative studies on anthocyanin synthesis in *S*. *oblata*.

### Transcriptional regulation of floral scent biosynthesis in *S*. *oblata*

In contrast to the rapid progress in recent years in characterizing and mapping the reactions leading to the formation of floral scent, little is known about regulation of floral scent production at the molecular level. The genetic basis and functional significance of scent production have also been investigated in petunia. To date, ODORANT1 (ODO1) is the first transcription factor that has been characterized as a regulator of scent production in flowers[62]. ODO1 belongs to the R2R3-MYB transcription factor family (subgroup with AtMYB42 and AtMYB85), and its suppression in *P*. *hybrida* leads to decreased levels of emitted volatile phenylpropanoids[62, 63]. The importance of regulating flux toward phenylpropanoids is also demonstrated in *P*. *hybrida* flowers over-expressing the *A*. *thaliana* MYB factor PRODUCTION OF ANTHOCYANIN PIGMENT1 (PAP1)[64]. Recently, another two transcription factors, EMISSION OF BENZENOIDS I and II (EOBI and EOBII), belonging to subgroup of R2R3-MYB family (subgroup 19), are also demonstrated to regulate benzenoid biosynthesis in petunias[65, 66]. Both *EOBI* and *EOBII* positively regulate *ODO1*, which determines the floral scent production in *P*. *hybrida*. However, the regulating mechanism of transcription factors the terpene has not yet been reported. In this study, we found that two R2R3-MYB (contigs_28146 and 38962) transcription factors were up-regulated at the SOB and SOF stages in *S*. *oblata* (S6 File). However, the molecular regulation mechanism of floral scent biosynthesis depending on MYBs still needs further research.

## Conclusion

The combination of RNA-seq and DEGs analysis based on Illumina sequencing technology provided comprehensive information on gene expression in *S*. *oblata*. In this study, we revealed numerous differentially expressed genes between different flowering stages. Candidate genes for biosynthesis of anthocyanin, flavonoids, flavones and flavonols, terpenoids, and monoterpenoids and for degradation of limonene and pinene were rapidly identified by this approach. In summary, this comprehensive database provides essential information for investigating flowering and other biological pathways in *S*. *oblata* and will be useful for improving the horticultural and ornamental quality of this species.

## Supporting Information

S1 Fig KOG classification of DEGs between SOB and SOFB. (TIF)

S2 Fig KOG classification of DEGs between SOF and SOB. (TIF)

S3 Fig KOG classification of DEGs between SOF and SOFB. (TIF)

S4 Fig Real-time qPCR validation of genes involved in flower pigmentation and scent biosynthesis in *S*. *oblata*. The y-axis indicates fold change in expression among SOFB (blue bars), SOB (red bars) and SOF (green bars) samples using the results of RT-qPCR. Data were normalized against the reference S. oblata ACT-7 gene. Quantitative PCR for each gene was performed with three biological replicates, with three technical replicates per experiment. Bars indicate SD.
(TIF)

S1 File Primers used for analysis of gene expression by qRT-PCR. (XLSX)

S2 File Summary of GO term assignment for the *S*. *oblata* inflorescence transcriptome (Level2). (XLSX)

S3 File KOG annotation of *S*. *oblata* unigenes. (XLSX)

S4 File Summary of KEGG pathways involved in the *S*. *oblata* inflorescence transcriptome. (XLSX)

S5 File Summary of flower pigment biosynthesis and floral scent metabolism in *S*. *oblata*. (XLSX)

S6 File Functional annotations and statistics for all DEGs. (XLSX)

S7 File GO classification of DEGs between SOB and SOFB. (PDF)

S8 File Comparison of gene set enrichment between SOB and SOFB by GO and KEGG. (XLSX)

S9 File GO classification of DEGs between SOF and SOB. (PDF)

S10 File Comparison of gene set enrichment between SOF and SOB by GO and KEGG. (XLSX)

S11 File GO classification of DEGs between SOF and SOFB. (PDF)

S12 File Comparison of gene set enrichment between SOF and SOFB by GO and KEGG. (XLSX)

S13 File Functional annotation of unigenes related to flower pigment biosynthesis and floral scent metabolism in *S*. *oblata* DEGs. (XLSX)

## References

[1] Huang JX, Wang LS, Li XM, Lu YQ. Advances in molecular basis and evolution of floral color variation. Chin Bull Bot. 2006, 23(4): 321–333.

[2] Shirley BW, Kubasek WL, Storz G, Bruggemann E, Koornneef M, Ausubel FM, et al Analysis of *Arabidopsis* mutants deficient in flavonoid biosynthesis. Plant J. 2002, 8(5): 659–671. 10.1046/j.1365-313x.1995.08050659.x 8528278

[3] Grotewold E. The genetics and biochemistry of floral pigments. Annu Rev Plant Biol. 2006, 57: 761–780. 16669781 10.1146/annurev.arplant.57.032905.105248

[4] Tanaka Y, Sasaki N, Ohmiya A. Biosynthesis of plant pigments: anthocyanins, betalains and carotenoids. Plant J. 2008, 54(4): 733–749. 10.1111/j.1365-313X.2008.03447.x 18476875

[5] Albert NW, Lewis DH, Zhang H, Schwinn KE, Jameson PE, Davies KM. Members of an R2R3-MYB transcription factor family in Petunia are developmentally and environmentally regulated to control complex floral and vegetative pigmentation patterning. Plant J. 2011, 65(5): 771–784. 10.1111/j.1365-313X.2010.04465.x 21235651

[6] Baudry A, Heim MA, Dubreucq B, Caboche M, Weisshaar B, Lepiniec L. TT2, TT8, and TTG1 synergistically specify the expression of BANYULS and proanthocyanidin biosynthesis in *Arabidopsis thaliana* . Plant J. 2004, 39(3): 366–380. 15255866 10.1111/j.1365-313X.2004.02138.x

[7] Elomaa P, Uimari A, Mehto M, Albert VA, Laitinen RAE, Teeri TH. Activation of anthocyanin biosynthesis in *Gerbera hybrida* (Asteraceae) suggests conserved protein-protein and protein-promoter interactions between the anciently diverged monocots and eudicots. Plant Physiol. 2003, 133(4): 1831–1842. 14605235 10.1104/pp.103.026039 PMC300736

[8] Nakatsuka T, SanaeHaruta K, Pitaksutheepong C, Abe Y, Kakizaki Y, Yamamoto K, et al Identification and characterization of R2R3-MYB and bHLH transcription factors regulating anthocyanin biosynthesis in gentian flowers. Plant Cell Physiol. 2008, 49(12): 1818–1829. 10.1093/pcp/pcn163 18974195

[9] Yamagishi M, Shimoyamada Y, Nakatsuka T, Masuda K. Two R2R3-MYB genes, homologs of petunia AN2, regulate anthocyanin biosyntheses in flower tepals, tepal spots and leaves of Asiatic hybrid lily. Plant Cell Physiol. 2010, 51(3): 463–474. 10.1093/pcp/pcq011 20118109

[10] LI Z, CAO H, LIU L, LI B. Chemical constituents of aroma in fresh *Syringa oblata* flowers. J Zhejiang For Coll. 2006, 23(2): 159–162.

[11] Hendel-Rahmanim K, Masci T, Vainstein A, Weiss D. Diurnal regulation of scent emission in rose flowers. Planta. 2007, 226(6): 1491–1499. 17636322 10.1007/s00425-007-0582-3

[12] Zhao J, Hu ZH, Leng PS, Zhang HX, Cheng FY. Fragrance composition in six tree peony cultivars. Kor J Hortic Sci Technol. 2012, 30(6): 617–625.

[13] Zhang HX, Hu ZH, Leng PS, Wang WH, Xu F, Zhao J. Qualitative and quantitative analysis of floral volatile components from different varieties of *Lilium spp* . Scientia Agri Sin. 2013, 46(4): 790–799.

[14] Hao RJ, Zhang Q, Yang WR, Wang J, Cheng TR, Pan HT, et al Emitted and endogenous floral scent compounds of *Prunus mume* and hybrids. Biochem System Ecol. 2014, 54: 23–30.

[15] Knudsen JT, Roger E, Jonathan G, Stahl B. Diversity and distribution of floral scent. Bot Rev. 2006, 72(1): 1–120.

[16] Nagegowda DA. Plant volatile terpenoid metabolism: Biosynthetic genes, transcriptional regulation and subcellular compartmentation. FEBS Lett. 2010, 584: 2965–2973. 10.1016/j.febslet.2010.05.045 20553718

[17] Cui HX, Jiang GM, Zang SY. The distribution, origin and evolution of *Syringa* . Bull Bot Res. 2004, 24(2): 141–145.

[18] Zang SY, Cui HX. lilac. Shanghai: Shanghai Science and Technology Press 2000.

[19] Li YN, Svenson SE. Syringa oblata. Am Nurserym. 2014, 214(2): 38.

[20] Wang Z, Gerstein M, Snyder M. RNA-Seq: a revolutionary tool for transcriptomics. Nature Rev Genet. 2009, 10(1): 57–63. 10.1038/nrg2484 19015660 PMC2949280

[21] 'tHoen PAC, Ariyurek Y, Thygesen HH, Vreugdenhil E, Vossen RHAM, Menezes ReXd, et al Deep sequencing-based expression analysis shows major advances in robustness, resolution and inter-lab portability over five microarray platforms. Nucleic Acids Res. 2008, 36(21): e141 10.1093/nar/gkn705 18927111 PMC2588528

[22] Liu DF, Sui SZ, Ma J, Li ZN, Guo YL, Luo DP, et al Transcriptomic analysis of flower development in wintersweet (*Chimonanthus praecox*). PLoS ONE. 2013, 9(1): e86976. 10.1371/journal.pone.0086976 PMC3906103 24489818

[23] Zhang JX, Wu KL, Zeng SJ, Silva JATd, Zhao XL, Tian CE, et al Transcriptome analysis of *Cymbidium sinense* and its application to the identification of genes associated with floral development. BMC Genomics. 2013, 14: 279 10.1186/1471-2164-14-279 23617896 PMC3639151

[24] Li XB, Luo J, Yan TL, Xiang L, Jin F, Qin DH, et al Deep sequencing-based analysis of the *Cymbidium ensifolium* floral transcriptome. PLoS ONE. 2013, 8(12): e85480 10.1371/journal.pone.0085480 24392013 PMC3877369

[25] Ge XX, Chen HW, Wang HL, Shi AP, Liu KF. De Novo assembly and annotation of *Salvia splendens* transcriptome using the Illumina platform. PLoS ONE. 2014, 9(3): e87693 10.1371/journal.pone.0087693 24622329 PMC3951189

[26] Robinson MD, McCarthy DJ, Smyth GK. edgeR: a Bioconductor package for differential expression analysis of digital gene expression data. Bioinformatics. 2010, 26(1): 139–140. 10.1093/bioinformatics/btp616 19910308 PMC2796818

[27] Anders S, Huber W. Differential expression analysis for sequence count data. Genome Biol. 2010, 11: R106 10.1186/gb-2010-11-10-r106 20979621 PMC3218662

[28] Su CL, Chao YT, Alex-Chang YC, Chen WC, Chen CY, Lee AY, et al De novo assembly of expressed transcripts and global analysis of the phalaenopsis aphrodite transcriptome. Plant Cell Physiol. 2011, 52 (9): 1501–1514. 10.1093/pcp/pcr097 21771864

[29] Garg R, Patel RK, Tyagi AK, Jain M. De novo assembly of chickpea transcriptome using short reads for gene discovery and marker identification. DNA Res. 2011, 18(1): 53–63. 10.1093/dnares/dsq028 21217129 PMC3041503

[30] Bräutigam A, Mullick T, Schliesky S, Weber APM. Critical assessment of assembly strategies for non-model species mRNA-Seq data and application of next-generation sequencing to the comparison of C3 and C4 species. J Exp Bot. 2011, 62(9): 3093–3012. 10.1093/jxb/err029 21398430

[31] Huang XQ, Madan A. CAP3: A DNA sequence assembly program. Genome Res. 1999, 9: 868–877. 10508846 10.1101/gr.9.9.868 PMC310812

[32] Pruitt KD, Tatusova T, Maglott DR. NCBI reference sequences (RefSeq): a curated non-redundant sequence database of genomes, transcripts and proteins. Nucleic Acids Res. 2007, 35(suppl. 1): D61–D65. 17130148 10.1093/nar/gkl842 PMC1716718

[33] Ashburner M, Ball CA, Blake JA, Botstein D, Butler H, Cherry JM, et al Gene ontology: tool for the unification of biology. The Gene Ontology Consortium. Nat Genet. 2000, 25(1): 25–34. 10802651 10.1038/75556 PMC3037419

[34] Moriya Y, Itoh M, Okuda S, Yoshizawa AC, Kanehisa M. KAAS: an automatic genome annotation and pathway reconstruction server. Nucleic Acids Res. 2007, 35(suppl. 2): 182–185. 10.1093/nar/gkm321 PMC1933193 17526522

[35] Young MD, Wakefield MJ, Smyth GK, Oshlack A. Gene ontology analysis for RNA-seq: accounting for selection bias. Genome Biol. 2010, 11: R14 10.1186/gb-2010-11-2-r14 20132535 PMC2872874

[36] Mao XZ, Cai T, Olyarchuk JG, Wei LP. Automated genome annotation and pathway identification using the KEGG Orthology (KO) as a controlled vocabulary. Bioinformatics. 2005, 21(19): 3787–3793. 15817693 10.1093/bioinformatics/bti430

[37] Cheadle C, Vawter MP, Freed WJ, Becker KG. Analysis of microarray data using Z score transformation. J Mol Diagn. 2003, 5(2): 73–81. 12707371 10.1016/S1525-1578(10)60455-2 PMC1907322

[38] Novaes E, Drost DR, Farmerie WG, Pappas GJ, Grattapaglia D, Sederoff RR, et al High-throughput gene and SNP discovery in *Eucalyptus grandis*, an uncharacterized genome. BMC Genomics. 2008, 9: 312 10.1186/1471-2164-9-312 18590545 PMC2483731

[39] Iwashina T, Konta F, Kitajima J. Anthocyanins and flavonols of *Chimonanthus praecox* (Calycanthaceae) as flower pigments. J Jap Bot. 2001, 76(3): 166–172.

[40] Hui RH, Li TC, Hou DY. Analysis of volatile componentsfrom flower and leaf of *syringa oblata* Lindl. By GC/MS. J Chin Mass Spectr Soc. 2002, 23(4): 210–213.

[41] Feng C, Chen M, Xu CJ, Bai L, Yin XR, Li X, et al Transcriptomic analysis of Chinese bayberry (*Myrica rubra*) fruit development and ripening using RNA-Seq. BMC Genomics. 2012, 13:19 10.1186/1471-2164-13-19 22244270 PMC3398333

[42] Liu MY, Qiao GR, Jiang J, Yang HQ, Xie LH, Xie JZ, et al Transcriptome sequencing and de novo analysis for Ma Bamboo (*Dendrocalamus latiflorus* Munro) using the Illumina platform. PLoS ONE. 2012, 7(10): e46766 10.1371/journal.pone.0046766 23056442 PMC3463524

[43] Li DJ, Deng Z, Qin B, Liu XH, Men ZH. De novo assembly and characterization of bark transcriptome using Illumina sequencing and development of EST-SSR markers in rubber tree (*Hevea brasiliensis* Muell. Arg.). BMC Genomics. 2012, 13: 192 10.1186/1471-2164-13-192 22607098 PMC3431226

[44] Deng MH, Wen JF, Huo JL, Zhu HS, Dai XZ, Zhang ZQ, et al Cloning and characterization of two novel purple pepper genes (*CHS* and *F3H*). Afr J Biotechnol. 2012, 11(9): 2389–2397.

[45] Kunu W, Thanonkeo S, Thanonkeo P. Cloning and expression analysis of dihydroxyflavonol 4-reductase (DFR) in *Ascocenda* spp. Afr J Biotechnol. 2012, 11(64): 12702–12709.

[46] Ogata J, Itoh Y, Ishida M, Yoshida H, Ozeki Y. Cloning and heterologous expression of cDNAs encoding flavonoid glucosyltransferases from *Dianthus caryophyllus* . Plant Biotechnol. 2004, 21(5): 367–375.

[47] Hatayama M, Ono E, Yonekura-Sakakibara K, Tanaka Y, Nishino T, Nakayama T. Biochemical characterization and mutational studies of a chalcone synthase from yellow snapdragon (*Antirrhinum majus*) flowers. Plant Biotechnol. 2006, 23: 273–378.

[48] Zhou L, Wang Y, Peng Z. Molecular characterization and expression analysis of chalcone synthase gene during flower development in tree peony (*Paeonia suffruticosa*). Afr J Biotechnol. 2011, 10(8): 1275–1284.

[49] Cardoso S, Lau W, Dias JE, Fevereiro P, Maniatis N. A Candidate-gene association study for berry colour and anthocyanin content in *Vitis vinifera* L. PLoS ONE. 2012, 7(9): e46021 10.1371/journal.pone.0046021 23029369 PMC3461038

[50] Togami J, Okuhara H, Nakamura N, Ishiguro K, Hirose C, Ochiai M, et al Isolation of cDNAs encoding tetrahydroxychalcone 2'-glucosyltransferase activity from carnation, cyclamen, and catharanthus. Plant Biotechnol. 2011, 28: 231–238.

[51] Pelletier MK, Murrell JR, Shirley BW. Characterization of flavonol synthase and leucoanthocyanidin dioxygenase genes in Arabidopsis. Further evidence for differential regulation of "early" and "late" genes. Plant Physiol. 1997, 113(4): 1437–1445. 9112784 10.1104/pp.113.4.1437 PMC158268

[52] Pelletier MK, Shirley BW. Analysis of flavanone 3-hydroxylase in *Arabidopsis* seedlings-Coordinate regulation with chalcone synthase and chalcone isomerase. Plant physiol. 1996, 111(1): 339–345. 8685272 10.1104/pp.111.1.339 PMC157841

[53] Nakatsuka A, Lzumi Y, Yamagishi M. Spatial and temporal expression of chalcone synthase and dihydroflavonol 4-reductase genes in the Asiatic hybrid lily. Plant Sci. 2003, 165(4): 759–767.

[54] Nakatsuka T, Nishihara M, Mishiba K, Yamamura S. Temporal expression of flavonoid biosynthesis-related genes regulates flower pigmentation in gentian plants. Plant Sci. 2005, 168(5): 1309–1318.

[55] Hu K. Expression of genes on anthocyanin biosythesis pathway control flower coloration in *Chrysanthemum* and *Cineraria* (PhD thesis). Beijing: Beijing Forestry University. 2010, 63–74.

[56] Dudareva N, Martin D, Kish CM, Kolosova N, Gorenstein N, Fäldt J, et al *(E)*-*β*-ocimene and myrcene synthase genes of floral scent biosynthesis in snapdragon: function and expression of three terpene synthase genes of a new terpene synthase subfamily. Plant Cell. 2003, 15(5): 1227–1241. 12724546 10.1105/tpc.011015 PMC153728

[57] Muhlemann JK, Maeda H, Chang C-Y, Miguel PS, Baxter I, Cooper bB, et al Developmental changes in the metabolic network of snapdragon flowers. PloS ONE. 2012, 7(7): e40381 10.1371/journal.pone.0040381 22808147 PMC3394800

[58] Dudareva N, Cseke L, Blanc VM, Pichersky E. Evolution of floral scent in *Clarkia*: novel patterns of S-Linalool synthase gene expression in the *C*. *breweri* flower. Plant Cell. 1996, 8(7): 1137–1148. 8768373 10.1105/tpc.8.7.1137 PMC161191

[59] Chen F, Tholl D, D'Auria JC, Farooq A, Pichersky E, Gershenzon J. Biosynthesis and emission of terpenoid volatiles from *Arabidopsis* flowers. Plant Cell. 2003, 15(2): 481–494. 12566586 10.1105/tpc.007989 PMC141215

[60] Guitton Y, Nicolè F, Moja S, Valot N, Legrand S, Jullien F, et al Differential accumulation of volatile terpene and terpene synthase mRNAs during lavender (*Lavandula angustifolia* and *L*. x *intermedia*) inflorescence development. Physiol Plantarum. 2010, 138(2): 150–163. 10.1111/j.1399-3054.2009.01315.x 20002329

[61] Feng LG, Chen C, Li TL, Wang M, Tao J, Zhao DQ, et al Flowery odor formation revealed by differential expression of monoterpene biosynthetic genes and monoterpene accumulation in rose (*Rosa rugosa* Thunb.). Plant Physiol Biochem. 2014, 75: 80–88. 10.1016/j.plaphy.2013.12.006 24384414

[62] Verdonk JC, Haring MA, Tunen AJv, Schuurink RC. *ODORANT1* regulates fragrance biosynthesis in petunia flowers. Plant Cell. 2005, 17(5): 1612–1624. 15805488 10.1105/tpc.104.028837 PMC1091778

[63] Yuan YW, Byers KJRP, HDB Jr. The genetic control of flower-pollinator specificity. Curr Opin Plant Biol. 2013, 16(4): 422–428. 10.1016/j.pbi.2013.05.004 23763819 PMC3748206

[64] Ben ZM, Negre-Zakharov F, Masci T, Ovadis M, Shklarman E, Ben-Meir H, et al Interlinking showy traits: co-engineering of scent and colour biosynthesis in flowers. Plant Biotechnol J. 2008, 6(4): 403–415. 10.1111/j.1467-7652.2008.00329.x 18346094

[65] Spitzer-Rimon B, Marhevka E, Barkai O, Marton I, Edelbaum O, Masci T, et al EOBII, a gene encoding a flower-specific regulator of phenylpropanoid volatiles' biosynthesis in petunia. Plant Cell. 2010, 22(6): 1961–1976. 10.1105/tpc.109.067280 20543029 PMC2910970

[66] Spitzer-Rimon B, Farhi M, Albo B, Cna'ani A, Zvi MMB, Masci T, et al The R2R3-MYB-like regulatory factor EOBI, acting downstream of EOBII, regulates scent production by activating ODO1 and structural scent-related genes in petunia. Plant Cell. 2012, 24(12): 5089–5105. 10.1105/tpc.112.105247 23275577 PMC3556977

