# Supplementary figures and images for: Transcriptome Analysis of Syringa oblata Lindl. Inflorescence Identifies Genes Associated with Pigment Biosynthesis and Scent Metabolism
Source: PLoS One. 2015 Nov 20;10(11):e0142542. doi: 10.1371/journal.pone.0142542 (PMC4654506; doi:10.1371/journal.pone.0142542)

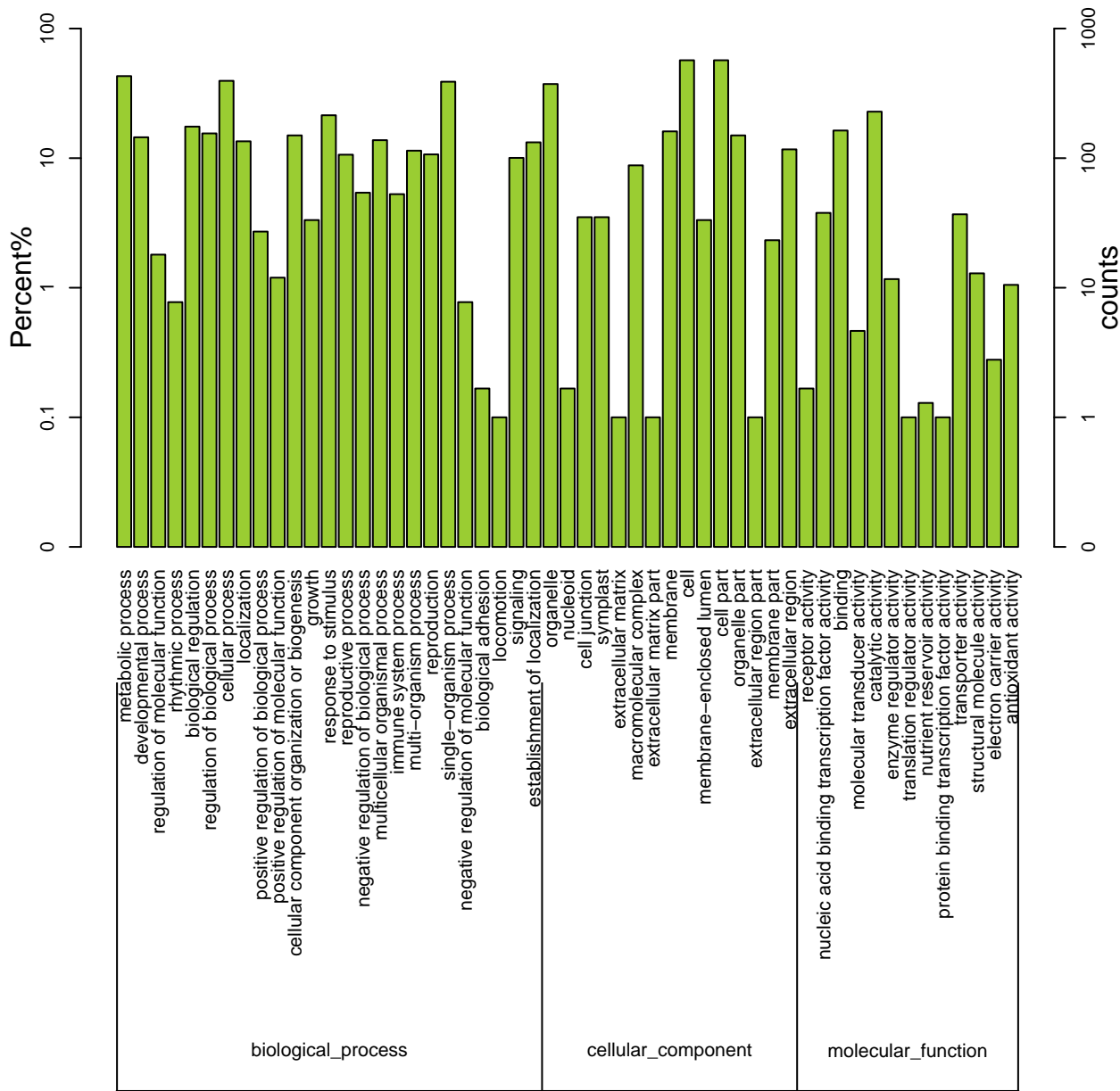

Supplement: S7 File — (PDF) [file pone.0142542.s011.pdf]

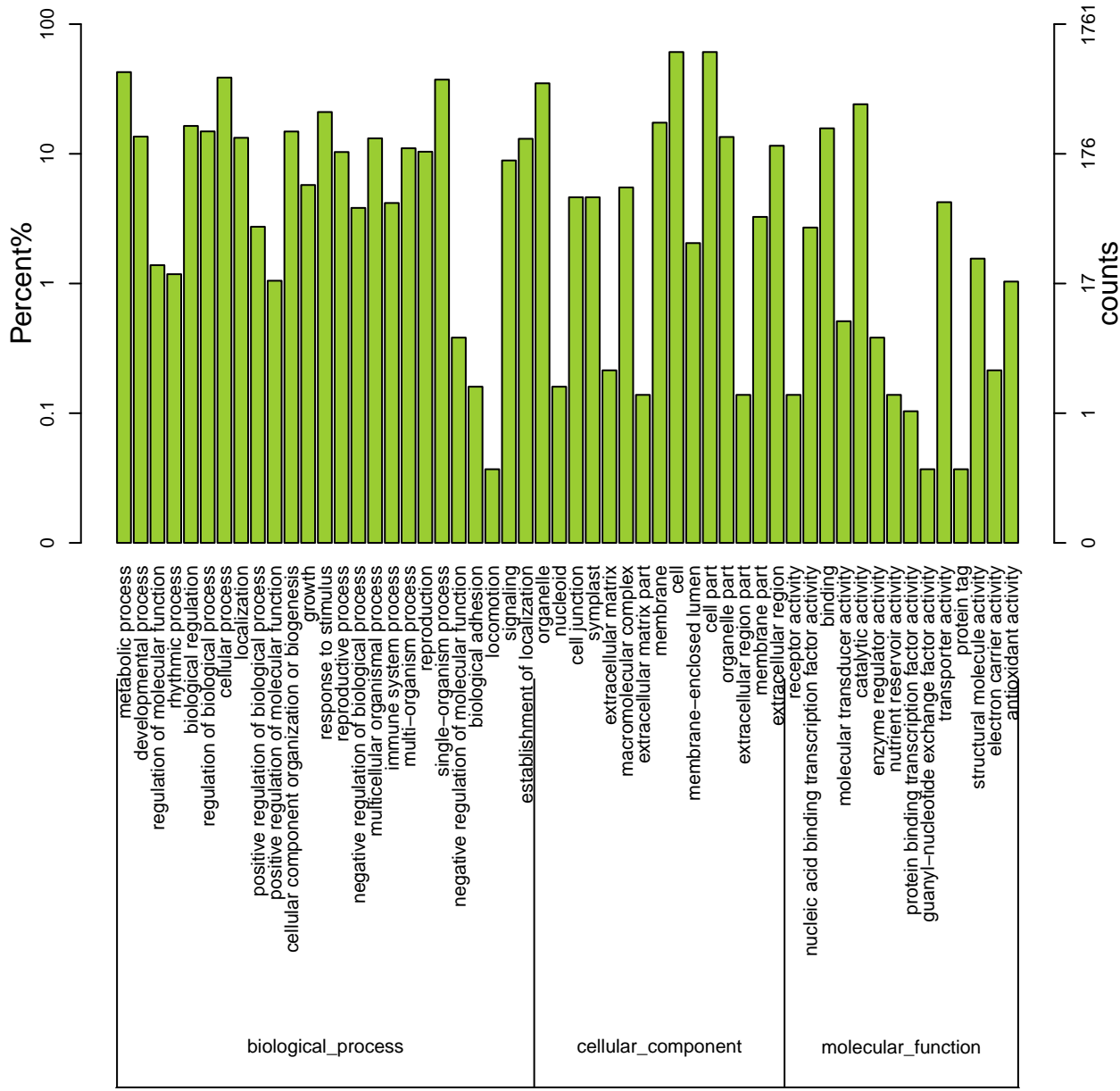

Supplement: S9 File — (PDF) [file pone.0142542.s013.pdf]

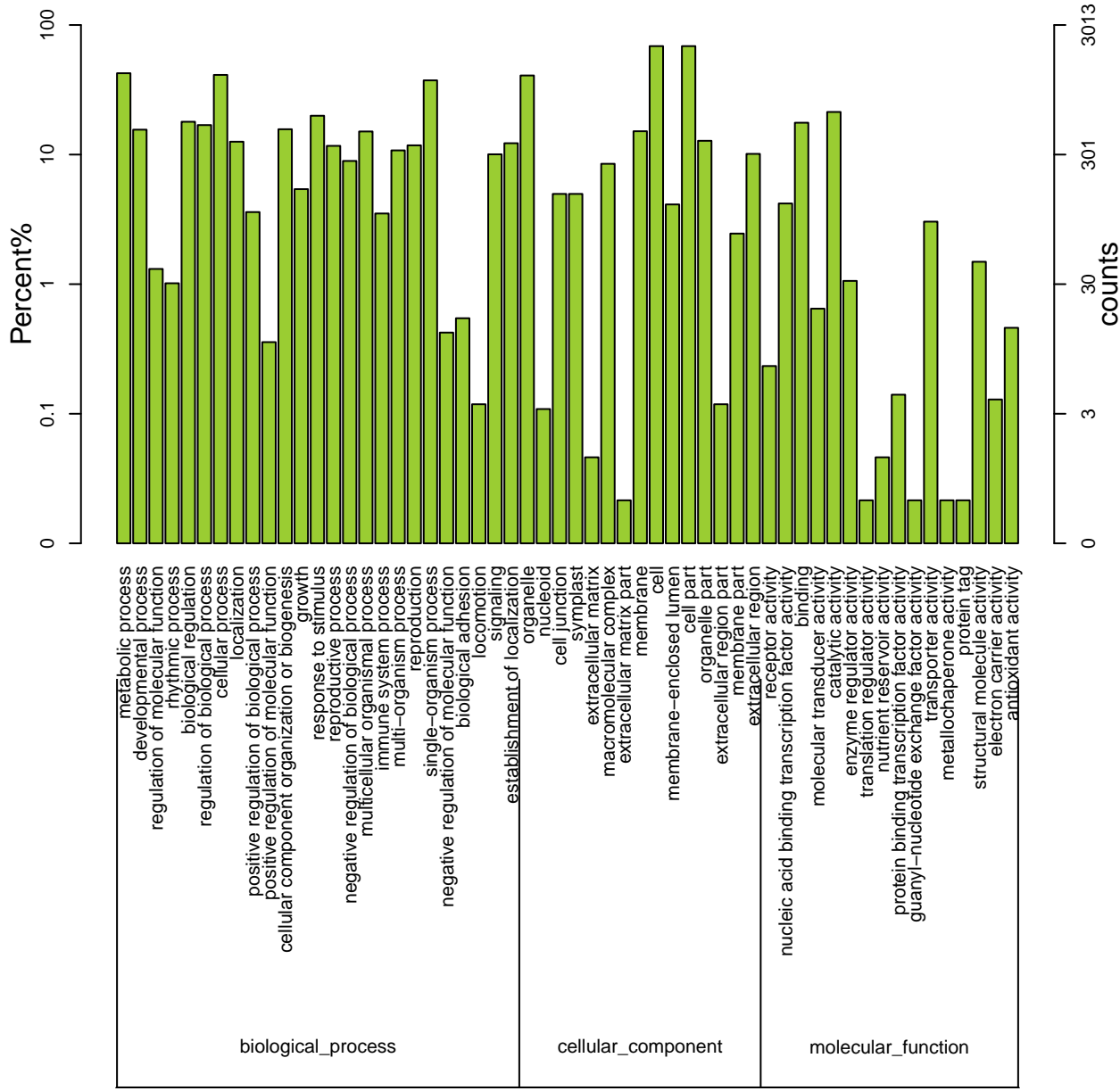

Supplement: S11 File — (PDF) [file pone.0142542.s015.pdf]
